# Influences of bacterial extracellular vesicles on macrophage immune functions

**DOI:** 10.3389/fcimb.2024.1411196

**Published:** 2024-05-30

**Authors:** Bowei Jiang, Junyun Huang

**Affiliations:** ^1^The First School of Clinical Medicine, Gannan Medical University, Ganzhou, China; ^2^Department of Laboratory Medicine, First Affiliated Hospital of Gannan Medical University, Ganzhou, China

**Keywords:** extracellular vesicles, bacterial, macrophage, immune, influences

## Abstract

Bacterial extracellular vesicles (EVs) are crucial mediators of information transfer between bacteria and host cells. Macrophages, as key effector cells in the innate immune system, have garnered widespread attention for their interactions with bacterial EVs. Increasing evidence indicates that bacterial EVs can be internalized by macrophages through multiple pathways, thereby influencing their immune functions. These functions include inflammatory responses, antimicrobial activity, antigen presentation, and programmed cell death. Therefore, this review summarizes current research on the interactions between bacterial EVs and macrophages. This will aid in the deeper understanding of immune modulation mediated by pathogenic microorganisms and provide a basis for developing novel antibacterial therapeutic strategies.

## 1 Introduction

Intercellular communication is an indispensable process in living systems, with various cells transmitting information and coordinating behavior by releasing extracellular signaling molecules. Bacteria, as single-celled organisms, exchange information with other bacteria and host cells by secreting extracellular vesicles (EVs), thereby participating in the regulation of various physiological and pathological processes (Tiku and Tan, 2021). Bacterial EVs are small membrane vesicles rich in bioactive molecules such as proteins, lipids, and nucleic acids, capable of serving as carriers for signaling molecules and playing crucial roles in biofilm formation, stress responses, quorum sensing, and nutrient acquisition (Kim et al., 2015; Huang et al., 2023).

In recent years, research has revealed that bacterial EVs not only affect the behavior of bacteria themselves but also play significant roles in the interactions between microbes and hosts, especially impacting the host immune system (Kaparakis-Liaskos and Ferrero, 2015). As the main effector cells of the innate immune system, the interactions between macrophages and bacterial EVs have garnered considerable attention. Macrophages recognize, phagocytose, and digest invading pathogens, playing a frontline role in immune defense (Locati et al., 2020). However, how various molecular signals carried by bacterial EVs affect the immune functions of macrophages remains a hot and challenging field. On one hand, certain pathogenic bacteria can interfere with and evade macrophage immune surveillance through EVs (Kuehn and Kesty, 2005; Bielaszewska et al., 2017). on the other hand, commensal bacteria can enhance macrophage antimicrobial capacity via EVs, playing a role in regulating host-microbe balance (Shen et al., 2012). Additionally, bacterial EVs can influence other physiological processes of macrophages, such as metabolic state and cell death (Dhital et al., 2021). Therefore, fully elucidating the molecular mechanisms of the interactions between bacterial EVs and macrophages is of great significance and value for understanding the immune regulatory networks mediated by microbes.

## 2 Types and biogenesis of bacterial EVs

First, compared to EVs produced by eukaryotic cells, bacterial EVs exhibit more diverse types and biogenesis mechanisms. Taking Gram-negative bacteria as an example, current research indicates that they can produce EVs through two mechanisms: blebbing and explosive cell lysis (Toyofuku et al., 2023). Specifically, EVs produced through blebbing include outer membrane vesicles (OMVs) and outer-inner membrane vesicles (OIMVs). OMVs are formed due to the disturbance in the Gram-negative bacterial cell envelope, leading to the outer membrane blebbing outward and eventually shedding off (Kadurugamuwa et al., 1993; Kulp and Kuehn, 2010; Schwechheimer and Kuehn, 2015). In contrast, OIMVs are formed when the peptidoglycan layer of Gram-negative bacteria undergoes autolysin-mediated weakening, resulting in the outer and inner membranes blebbing outward together (Perez-Cruz et al., 2013; Perez-Cruz et al., 2015). Additionally, Gram-negative bacteria can produce explosive outer membrane vesicles (EOMVs) and explosive outer-inner membrane vesicles (EOIMVs) through the explosive cell lysis mechanism. This occurs when phage-encoded endolysins in Gram-negative bacteria are activated, degrading the peptidoglycan layer and causing explosive cell lysis, followed by spontaneous assembly and fusion of the fragmented membrane segments (Turnbull et al., 2016; Baeza et al., 2021).

As for Gram-positive bacterial EVs, current research indicates that the peptidoglycan cell wall of Gram-positive bacteria can form pores under the action of endolysins, autolysins, or certain antibiotics that inhibit peptidoglycan synthesis, ultimately leading to the cytoplasmic membrane protruding outward and forming cytoplasmic membrane vesicles (CMVs) (Mitchell et al., 2013; Toyofuku et al., 2017; Andreoni et al., 2019; Abe et al., 2021; Liu et al., 2022). However, throughout this process, Gram-positive bacterial cells do not undergo explosive lysis. Nonetheless, the disruption of cytoplasmic membrane integrity still results in bacterial cell death. Therefore, this mechanism is termed “bubbling cell death” (Jiang et al., 2024).

## 3 Macrophage internalization of bacterial EVs

Endocytosis is the main pathway through which bacterial EVs enter macrophages. Endocytosis refers to the process by which small molecules cross the bilayer cell membrane and enter the cell (Doherty and McMahon, 2009). Due to the differences in the surface and cargo of bacterial EVs, the pathways of endocytosis can vary. For instance, *Staphylococcus aureus* EVs can be internalized by macrophages through a dynamin-dependent endocytic pathway (Wang et al., 2020). *Klebsiella pneumoniae* EVs can enter macrophages through a lectin-like oxidized low-density lipoprotein receptor-dependent endocytic pathway (Ye et al., 2021). Additionally, clathrin-mediated endocytosis is also closely related to the entry of bacterial EVs into macrophages. Clathrin-mediated endocytosis involves the formation of a clathrin-coated pit, which generates vesicles to absorb target substances (Vercauteren et al., 2010). This mode of endocytosis plays a crucial role in the uptake of external substances by host cells, but it is usually tightly regulated by cell surface receptors, allowing only substances that specifically bind to the receptors to enter the cell (McMahon and Boucrot, 2011; Kaksonen and Roux, 2018; Mettlen et al., 2018). However, most current studies on EV internalization pathways focus on Gram-negative bacteria. Future research could further investigate the pathways of Gram-positive bacteria EV internalization.

## 4 The impact of bacterial EVs on macrophage immune function

**Figure 1:**
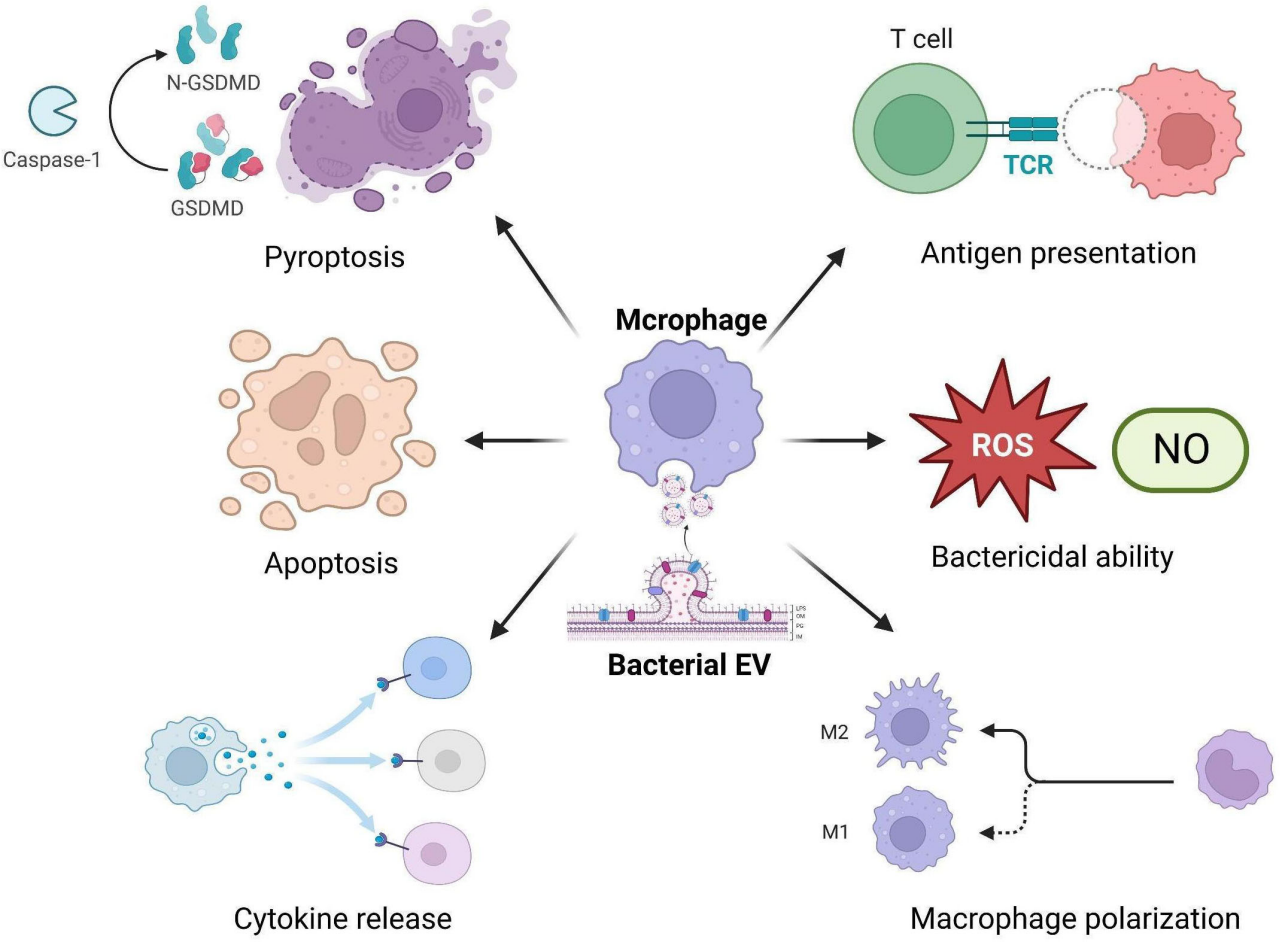
Bacterial EVs interact with macrophages and affect their immune functions.

Increasing evidence suggests that bacterial EVs can significantly impact the immune functions of macrophages, including inflammatory responses, antibacterial activity, antigen presentation capacity, and programmed cell death (Ellis et al., 2010; Dhital et al., 2021; Hu et al., 2021) (**Figure 1**).

### 4.1 Bacterial EVs can modulate macrophage-mediated inflammatory responses

First, when bacterial EVs come into contact with or are phagocytosed by macrophages, the pathogen-associated molecular patterns (PAMPs) they carry interact with the pattern recognition receptors (PRRs) of macrophages (Asano et al., 2021). This interaction induces macrophages to produce various inflammatory factors (Ellis et al., 2010; Asano et al., 2021; Xu et al., 2022). For example, lipoproteins in *S. aureus* EVs can stimulate macrophages to release IL-6 and TNF-α (Kopparapu et al., 2021). LPS and flagellin in *Pseudomonas aeruginosa* EVs can stimulate macrophages to release MIP-2, TNF-α, and IL-6 (Ellis et al., 2010). Extracellular RNA in *Aggregatibacter actinomycetemcomitans* EVs can promote TNF-α production in macrophages (Han et al., 2019). These examples show that bacterial EVs can significantly promote macrophage-mediated inflammatory responses. However, in addition to their pro-inflammatory effects, EVs from some bacteria, such as *Escherichia coli*, *Francisella tularensis*, and *Clostridium butyricum*, can stimulate macrophages to release the anti-inflammatory cytokine IL-10 (Hu et al., 2020; Pavkova et al., 2021; Guangzhang et al., 2023). This indicates that EVs from certain bacteria have complex dual effects on macrophage-mediated inflammatory responses, capable of inducing the production of both pro-inflammatory and anti-inflammatory cytokines by macrophages. Moreover, besides directly inducing the production of various cytokines, studies have found that EVs from *E. coli* can induce macrophages to release pro-inflammatory EVs after interacting with them. This implies that EVs derived from *E. coli* have the ability to transmit inflammatory signals to macrophage EVs (Imamiya et al., 2023). Interestingly, although *Porphyromonas gingivalis* EVs can stimulate macrophages to release various inflammatory cytokines, the Arg- and Lys-gingipains enzymes in these EVs can degrade macrophage inflammatory cytokines, thereby achieving fine-tuned regulation of the immune response (Barth and Genco, 2016; Castillo et al., 2022).

Additionally, it is important to note that not all bacterial EVs exhibit pro-inflammatory effects. Current research indicates that EVs produced by some commensal bacteria, such as *Lactobacillus plantarum*, *C. butyricum*, and *Bacteroides thetaiotaomicron*, can induce macrophages to polarize towards the M2 type, leading to the release of the anti-inflammatory cytokine IL-10, thereby inhibiting inflammatory responses (Kim et al., 2020; Muller et al., 2021; Liang et al., 2022). Moreover, studies have found that certain mycobacteria, such as *Mycobacterium avium* and *Mycobacterium kansasii*, also produce EVs with anti-inflammatory properties that can induce macrophages to produce IL-10 (Kamran-Sarkandi et al., 2018; Tavassol et al., 2020). Therefore, these studies demonstrate that EVs from different bacterial sources have varying effects on macrophage-mediated inflammatory responses.

### 4.2 Bacterial EVs can influence the antibacterial activity of macrophages

Additionally, as immune cells, macrophages possess strong antibacterial capabilities. When pathogens are recognized by macrophages, they form phagosomes on their cell membranes to engulf the pathogens, which then fuse with lysosomes to form phagolysosomes, thereby eliminating the pathogens (Hirayama et al., 2017). However, it is worth noting that bacterial EVs have varying effects on the antibacterial activity of macrophages. For example, Blancá et al (Blanca et al., 2022). investigated the effects of *Bordetella pertussis*-derived EVs on the antimicrobial activity of macrophages. The study found that *B. pertussis* EVs contain various toxins, including adenylate cyclase (CyaA). It was shown that pre-incubating macrophages with EVs significantly weakened their phagocytic and bactericidal activities against *B. pertussis*. This is because the CyaA in *B. pertussis* EVs inhibits the antibacterial capacity of macrophages by reducing the level of complement receptor 3 on their surface. Furthermore, Jung et al. (2016) studied how EVs from *Legionella pneumophila* affect the immune response of macrophages and promote bacterial replication within macrophages. Their research revealed that macrophages pre-treated with *L. pneumophila* EVs responded differently to subsequent Legionella infection. Initially, the EVs enhanced the immune defense response of macrophages and reduced bacterial replication. However, as the infection progressed, this effect gradually shifted, making macrophages more tolerant of bacterial replication. These findings suggest that *L. pneumophila* EVs have a complex dual role in macrophages, initially activating immune defenses and then creating an environment conducive to bacterial survival and replication. Interestingly, studies have shown that while EVs from *Streptococcus pneumoniae* can enhance macrophage phagocytosis of *S. pneumoniae*, this makes macrophages a “reservoir” for the bacteria, negatively impacting their antibacterial activity (Yerneni et al., 2021). On the other hand, bacterial EVs can also promote the antibacterial activity of macrophages. For example, EVs from *P. gingivalis* and *E. coli* can induce macrophages to produce reactive oxygen species (ROS) and nitric oxide (NO) upon interaction (Imayoshi et al., 2011; Guangzhang et al., 2023). These substances are important antibacterial weapons of macrophages, capable of exerting toxic effects directly on pathogens (Nathan and Hibbs, 1991; Canton et al., 2021; Herb and Schramm, 2021). Additionally, EVs from *E. coli* Nissle can increase the activity of acid phosphatase in macrophages (Hu et al., 2020). This enzyme is located in lysosomes and is closely related to the digestive functions of macrophages (Xu et al., 2012).

### 4.3 Bacterial EVs can influence the antigen-presentation function of macrophages

Additionally, macrophages possess important antigen-presenting functions. After pathogens are digested by macrophages, they are degraded into multiple small antigen fragments. These antigen fragments bind to major histocompatibility complex (MHC) molecules in macrophages, thereby triggering specific immune responses (Guerriero, 2019). However, it should be noted that some studies have indicated that EVs secreted by certain bacteria have the ability to affect the antigen-presenting function of macrophages. For example, Armstrong et al. (2020) investigated the impact of EVs secreted by *P. aeruginosa* on the expression of MHC-related molecules in human lung macrophages. The study found that *P. aeruginosa* EVs significantly inhibited the expression of 11 different MHC-related molecules in lung macrophages, including several subunits of MHC class II molecules (HLA-DRA, HLA-DPA1, etc.). Additionally, three CpG sites within the CFB gene showed significant hypomethylation under the influence of EVs, which was closely associated with the downregulation of MHC gene expression. These results suggest that *P. aeruginosa* EVs may regulate the antigen-presenting function of macrophages by altering the epigenetic state of their genes. Moreover, another study found that EVs from *E. coli* can promote the expression of the co-stimulatory molecule CD86 on the surface of macrophages (Guangzhang et al., 2023). This molecule plays a crucial role in the activation of T cells induced by macrophages (Smith et al., 2016; Nordlohne et al., 2021). However, current research on the impact of bacterial EVs on the antigen-presenting function of macrophages is still limited, and it has not been clearly identified which components in EVs interfere with this function. This area warrants further exploration in future research.

### 4.4 Bacterial EVs can induce programmed cell death in macrophages

Programmed cell death is an orderly and controlled process of cell death, which includes apoptosis, pyroptosis, and autophagy (Newton et al., 2024). First, pyroptosis is an inflammation-related form of cell death characterized by the release of cellular contents and the subsequent induction of an inflammatory response (Rao et al., 2022). Current studies indicate that bacterial EVs can deliver a series of bacterial molecules to macrophages to induce pyroptosis. For instance, *E. coli* EVs can transport LPS into the cytoplasm, directly activating caspase-11 to induce pyroptosis in macrophages (Vanaja et al., 2016; Finethy et al., 2017). Similarly, lipoproteins and pore-forming toxins in *S. aureus* EVs can activate caspase-1 through the NLRP3 inflammasome, thereby inducing pyroptosis in macrophages (Wang et al., 2020). Additionally, bacterial EVs containing flagellin can induce pyroptosis in macrophages by activating the NLRC4 inflammasome rather than the NLRP3 inflammasome (Bitto et al., 2018). Unlike the NLRP3 inflammasome, the NLRC4 inflammasome specifically recognizes bacterial flagellin and flagellin-like molecules, leading to a more specific and rapid activation (Gram et al., 2021; Chebly et al., 2022). For example, *Salmonella* EVs containing flagellin can quickly activate the NLRC4 inflammasome in a shorter time compared to flagellin-deficient *E. coli* and *Salmonella* EVs (Yang et al., 2020). Interestingly, *P. gingivalis* itself cannot activate inflammasome formation in macrophages, but its EVs can, as observed in a study by Fleetwood et al. (2017). This might be due to the enrichment of gingipain in *P. gingivalis* EVs, which helps *P. gingivalis* survive within cells (Sheets et al., 2008; Mantri et al., 2015). After being phagocytosed by macrophages, *P. gingivalis* downregulates gingipain levels (Xia et al., 2007). Moreover, the activation of inflammasomes in macrophages by bacterial EVs requires the involvement of guanylate-binding proteins (GBPs) (Finethy et al., 2017; Santos et al., 2018). GBPs are a class of large GTPases that play a crucial role in inflammasome activation (Shenoy et al., 2012).

Additionally, bacterial EVs can also induce apoptosis in macrophages. Apoptosis is a non-inflammatory form of cell death that can occur via intrinsic and extrinsic pathways (Kiraz et al., 2016). Initial studies using *Neisseria gonorrhoeae* EVs to treat bone marrow-derived macrophages found that the toxin protein PorB in N. gonorrhoeae EVs was sufficient to cause mitochondrial dysfunction in macrophages, leading to the release of cytochrome c and inducing apoptosis (Deo et al., 2018). However, recent research has discovered that EVs from *N. gonorrhoeae*, uropathogenic *E. coli*, and *P. aeruginosa* cause mitochondrial dysfunction in macrophages, which inhibits intracellular protein synthesis without inducing rapid apoptosis (Deo et al., 2020). Nonetheless, prolonged inhibition of protein synthesis can regulate members of the BCL-2 family, thereby activating mitochondrial apoptosis (Speir et al., 2016). Consequently, over time, EVs indirectly activate the pro-apoptotic member BAK, leading to apoptosis (Czabotar et al., 2014).

Moreover, autophagy, as a form of programmed cell death, is also an important antibacterial mechanism in macrophages (Wu and Lu, 2019). However, to date, only one study by David et al. has reported on the effects of bacterial EVs on macrophage autophagy. This study found that EVs produced by *E. coli* expressing HlyF can inhibit autophagic flux by preventing the fusion of autophagosomes with lysosomes, thereby hindering the formation of acidic autolysosomes and the clearance of autophagosomes. This suggests that EVs from *E. coli* expressing HlyF can enhance bacterial virulence by inhibiting autophagy in macrophages (David et al., 2022).

### 4.5 Bacterial EVs can influence the antiviral immune response of macrophages

It is worth noting that, in addition to the aforementioned impacts, current research indicates that bacterial EVs can also affect the antiviral immune response of macrophages. For instance, Bierwagen et al. (2023) explored the interaction between bacterial EVs and human macrophages and how this interaction influences viral replication, particularly concerning influenza virus. This study focused on EVs from different Gram-negative bacteria (such as *Klebsiella*, *E. coli*, and *Salmonella*) and found that these EVs can activate the antiviral immune response in macrophages via the TLR4-TRIF signaling axis. The research demonstrated that these bacterial EVs can induce macrophages to produce type I interferons and other antiviral molecules like Mx1, effectively inhibiting the replication of influenza virus A. Additionally, Bhar et al. (2022) investigated how commensal bacterial EVs influence murine norovirus (MNV) infection by modulating the antiviral immune response of macrophages. The study found that commensal bacterial EVs can bind to MNV and promote the co-inoculation of the virus and vesicles into host cells. This co-inoculation method significantly reduced viral replication in host cells compared to infection with the virus alone. Furthermore, these EVs increased the production of pro-inflammatory cytokines such as IL-6 and TNFα in macrophages during MNV infection, suggesting that EVs control viral infection by enhancing the immune response of host cells.

### 4.6 Bioengineered bacterial EVs can modulate the anti-tumor effects of macrophages

It is noteworthy that engineered bacterial EVs have also been reported to affect the antitumor immune effects of macrophages. Some tumor cells overexpress the CD47 molecule, which can inhibit the phagocytic function of macrophages, thus avoiding clearance (Kaur et al., 2016). Therefore, effectively activating tumor-associated macrophages is crucial in tumor therapy. In this regard, Feng et al. (2022) developed engineered bacterial OMVs designed to activate the phagocytic function of macrophages in the tumor microenvironment. These engineered vesicles were fused with CD47 nanobodies (CD47nb) on their surface and encapsulated with a layer of selenium-bonded polyethylene glycol (PEG/Se), enabling controlled release in the tumor site triggered by radiation. The results showed that OMV-CD47nb significantly enhanced the phagocytic ability of macrophages against tumor cells, primarily by inducing M1 polarization and blocking the “don’t eat me” signal on tumor cells. Additionally, Guo et al. (2021) developed a drug delivery system based on bacterial OMVs for sequential targeting of tumor cells and macrophages. This system utilized OMVs from Gram-negative bacteria loaded with paclitaxel (PTX) and siRNA targeting Redd1 in macrophages, achieving sequential drug release through a pH-sensitive linkage. The system first released PTX in the acidic environment of the tumor site, followed by siRNA delivery to M2 macrophages, enhancing glycolysis and promoting their polarization to the antitumor M1 type. The results showed that this OMV system demonstrated significant antitumor activity both *in vitro* and *in vivo*, effectively regulating macrophage metabolism and inhibiting tumor cell migration and invasion. These studies indicate that engineered bacterial EVs can achieve antitumor effects by influencing macrophages, providing new strategies for future cancer therapies.

## 5 Conclusion

Bacterial EVs, as crucial information carriers between bacteria and the host, have a profound impact on key immune cells in the host immune system, particularly macrophages. EVs from different bacterial sources can carry various bioactive molecules that regulate macrophage inflammatory responses, antibacterial activity, antigen presentation functions, and induce different forms of programmed cell death, thus reshaping microbe-host interactions at the microscopic level. On one hand, certain pathogenic bacterial EVs can stimulate macrophages to release pro-inflammatory factors, inhibit their bactericidal capabilities, interfere with antigen presentation functions, and induce cell death. On the other hand, some commensal bacterial EVs can induce macrophages to release anti-inflammatory factors, enhance bactericidal activity, and activate antiviral immune responses, thereby providing protective effects for the host. In addition to these impacts, engineered bacterial EVs can also modulate macrophage metabolic states and polarization types, influencing their antitumor immune effects. Moreover, they can serve as novel drug carriers targeting tumors and regulating macrophages, demonstrating significant potential in the field of cancer therapy.

## Author contributions

BJ: Writing – original draft. JH: Writing – review & editing.
